# Industrial Weld Defect Detection Based on Monocular Depth Estimation and Dual-Attention Point Cloud Network

**DOI:** 10.3390/s26113321

**Published:** 2026-05-23

**Authors:** Nannan Zhao, Shijie Chen

**Affiliations:** 1 School of Computer Science and Engineering, Guangdong Ocean University, Yangjiang 529568, China; 2 College of Electronic and Information Engineering, Guangdong Ocean University, Zhanjiang 524088, China; shijiec56@gmail.com

**Keywords:** weld defect detection, monocular depth estimation, pseudo-point cloud, dual attention mechanism, PointNet++, industrial quality inspection

## Abstract

In industrial quality control, the precise identification of severe structural weld defects is paramount. Traditional 2D image-based detection methods are susceptible to illumination and texture interference, while high-precision 3D laser scanning solutions are costly and impractical for large-scale deployment. To achieve reliable geometric defect detection at low cost, this paper proposes a detection framework based on monocular depth estimation and a dual-attention point cloud network. First, YOLOv8 is employed for rapid region of interest extraction, and an advanced monocular depth estimation model generates 3D pseudo-point clouds containing geometric information. Secondly, addressing the challenge of distinct spatial orientation features in missed weld defects that are prone to confusion, this paper introduces a dual-attention-enhanced point cloud classification network named DA-PointNet++. This model embeds dual-attention modules within the PointNet++ backbone network, enhancing key feature representation in both the channel and spatial dimensions. Experimental results demonstrate that this approach achieves an accuracy of 93.67% and a recall rate of 90.51% in a unified binary classification task for general weld defect detection, effectively identifying both normal welds and complex missed weld defects. Compared to PointConv, Dynamic Graph Convolutional Neural Network (DGCNN), and mainstream Point Cloud Transformer, this method significantly reduces false negative rates while maintaining low computational costs, offering a cost-effective solution for industrial automation.

## 1. Introduction

With the rapid advancement of Industry 4.0 and smart manufacturing, surface quality control for high-end equipment manufacturing has become a core element in ensuring product reliability [1,2]. Particularly in automated welding, recent review studies indicate that severe structural defects such as lack of fusion and missed welds frequently arise due to process parameter fluctuations or environmental thermal stresses, posing significant threats to structural integrity [3]. Traditional weld inspection primarily relies on manual visual inspection or physical testing, suffering from inefficiency, high subjectivity, and difficulties in online integration. In recent years, deep learning-based computer vision technology has gradually become the mainstream choice for industrial quality inspection due to its non-contact and high-speed advantages [4,5].

Current automated surface defect detection methods primarily fall into two categories: 2D image-based and 3D point cloud-based approaches. Methods utilising 2D convolutional neural networks, particularly single-stage object detectors such as the YOLO series, have gained widespread industrial adoption due to their exceptional real-time performance [6,7]. However, research by Vasan et al. indicates that 2D optical images are inherently two-dimensional projections of the three-dimensional world. They are highly susceptible to complex ambient lighting, oil contamination, and low-contrast surface textures, leading to high false alarm rates [8,9].

To overcome the limitations of 2D vision, researchers began incorporating auxiliary 3D geometric information. 3D sensors can directly capture the depth topology of object surfaces and are insensitive to lighting variations [10]. Although 3D methods offer significant advantages in detection accuracy, high-precision industrial-grade 3D scanning equipment is typically costly and demands stringent installation and calibration requirements. This severely limits its deployment in small and medium-sized enterprises and large-scale production lines. Consequently, acquiring and utilising high-precision 3D geometric features for defect detection without introducing expensive hardware has become a critical issue requiring urgent resolution.

Addressing these challenges, recent advances in computer vision—specifically monocular depth estimation—offer novel approaches for low-cost 3D perception. Godard et al.’s self-supervised depth estimation method demonstrated the feasibility of recovering geometric structures from a single image [11]. Subsequently, the pseudo-lidar concept introduced by Wang et al. further validated that converting depth maps into 3D point cloud representations can significantly enhance detection performance [12]. Notably, with the emergence of foundational models like Depth Anything, zero-shot depth estimation using large-scale data has achieved unprecedented accuracy and demonstrated its efficacy in geometric information recovery for autonomous driving perception tasks [13,14]. Inspired by this, this paper proposes a low-cost industrial defect detection framework based on pseudo-point cloud generation. It employs YOLOv8 for rapid region-of-interest localisation, combined with the Depth Anything model to generate high-fidelity pseudo-point clouds.

After acquiring 3D point cloud data, designing an efficient classification network presents another challenge. Whilst PointNet++ serves as a classic baseline, its max-pooling operation struggles to capture defects’ spatial directional features. Although Transformer-based models demonstrate strong performance across multiple point cloud tasks, their high computational complexity hinders direct application in industrial scenarios demanding real-time processing [15,16]. Inspired by lightweight network designs such as MobileNetV3 and ShuffleNet, we focus on enhancing feature extraction capabilities while maintaining a low computational load [17,18]. Furthermore, Guo et al. noted in their recent review of attention mechanisms that hybrid attention combining both channel and spatial dimensions most effectively captures key features [19]. To this end, inspired by the structural design philosophy of the Convolutional Block Attention Module (CBAM), this paper proposes an enhanced dual-attention point cloud network named DA-PointNet++ based on PointNet++. This network incorporates a lightweight dual-attention module within its Set Abstraction layer, significantly enhancing the model’s ability to distinguish missed weld defects across different orientations [20].

In summary, the principal contributions of this paper are outlined as follows:

Low-cost 2D–3D collaborative detection framework: A novel coarse-to-fine industrial defect detection paradigm is proposed. By integrating YOLOv8 with the Depth Anything model, pseudo-point cloud generation incorporating geometric structural information is achieved using monocular vision. This effectively eliminates reliance on expensive hardware such as high-precision 3D LiDAR sensors, substantially reducing industrial deployment costs.

Dual Attention Enhancement Mechanism: To address the loss of fine-grained features caused by PointNet++’s max-pooling operation, a lightweight dual-attention module is designed. This module significantly enhances the network’s perception and discrimination capabilities for structural defects with specific spatial orientations through adaptive feature recalibration across both channel and spatial dimensions.

Superior Performance: Extensive experiments were conducted on real-world welding defect datasets. Results demonstrate that the proposed method achieves an accuracy of 93.67% and a recall rate of 90.51%. Under equivalent computational conditions, this performance markedly surpasses mainstream point cloud models such as PointConv, DGCNN, and PCT, showcasing its substantial application potential in automated industrial quality inspection.

## 2. Related Work

Within both international academia and industry, automated surface defect detection is widely recognised as a core component of intelligent manufacturing. Deep learning approaches have been extensively applied in quality inspection for steel, textiles, and electronic components [1,4]. Addressing scenarios demanding high real-time performance, Liu et al. summarised deep learning-based real-time surface defect detection methods, highlighting the future trend towards balancing lightweight implementation with high accuracy [5]. Currently, single-stage detectors represented by the YOLO series are the mainstream choice. However, pure 2D methods lack robustness when confronted with complex lighting conditions or geometric defects lacking texture [6]. Recently, Vasan et al. proposed an ensemble learning-based defect classification model. While improving accuracy, it remains constrained by the inherent information gaps in 2D imagery [8]. Although multimodal fusion addresses this limitation, it typically relies on costly 3D sensors.

Unlike the regular arrangement of 2D pixels, 3D point clouds exhibit disorder and unstructured characteristics. Guo et al. comprehensively reviewed the latest advances in deep learning for 3D point clouds, categorising them into point-based, projection-based, and voxel-based methods [21]. PointNet and its enhanced variant PointNet++, proposed by Qi et al., represent milestones in point-based approaches [15,22]. Subsequently, the Dynamic Graph Convolutional Neural Network (DGCNN) enhanced through dynamic graph convolutions; PointConv extended continuous convolution operators [23,24]. Although Transformer architectures have set new benchmarks in 3D object detection and classification, their high computational cost limits edge-side deployment [25]. Given the industrial sector’s paramount pursuit of efficiency, this paper selects the computationally more efficient PointNet++ as the backbone network.

To deploy deep models on mobile and edge devices, lightweight network design has become a hot topic. Chen et al. provided a systematic review of lightweight Deep Convolutional Neural Networks (DCNNs), highlighting separable convolutions and channel shuffling as key techniques for reducing computational load [26]. Concurrently, attention mechanisms have demonstrated significant performance gains at minimal computational cost. Since the Squeeze-and-Excitation Network (SE-Net) pioneered through channel dependencies, attention mechanisms have gained widespread adoption in visual tasks [27]. For instance, coordinate attention embeds positional information into the channel dimension, demonstrating more efficient feature aggregation capabilities in lightweight mobile networks [28]. Guo et al. conducted an exhaustive classification study of attention mechanisms in computer vision, further validating their effectiveness in feature recalibration [19].

Alongside the introduction of attention mechanisms and lightweight designs, significant progress has been made in feature extraction and defect detection applications for three-dimensional point clouds. Jovančević et al. achieved automated detection and characterisation of surface defects on aircraft exteriors through 3D point cloud analysis, demonstrating the critical value of spatial geometric information in complex industrial quality inspection [29]. To enhance point cloud networks’ performance in handling complex spatial features, PointWeb proposes constructing local fully connected networks to augment contextual feature extraction within point cloud neighbourhoods [30]. Meanwhile, residual Multi-Layer Perceptron (MLP)-based frameworks offer lightweight paradigm for local geometric modelling [31]. Regarding global feature modelling, self-attention mechanisms incorporating Gumbel subset sampling and the recently proposed Point Transformer v3 have substantially enhanced the accuracy and generalisation capabilities of point cloud processing [32,33]. Furthermore, PointNeXt has re-tapped the immense potential of the PointNet++ backbone architecture through refined training strategies and model scaling [34]. In the front-end 3D geometric perception stage, Ranftl et al. achieved zero-shot cross-dataset transfer by blending multi-source datasets, markedly enhancing depth estimation robustness in complex environments [35]. Drawing upon these research advancements, this paper adopts lightweight and feature-enhancing design principles to devise a dual-attention module for point clouds. This achieves improved perception of minute geometric defects with minimal parametric overhead.

Monocular depth estimation aims to reconstruct 3D structures from a single 2D image, representing a key technology for reducing 3D perception costs. Godard et al.’s Monodepth2 employs self-supervised learning to address the challenge of obtaining labelled data [11]. Afshar et al. further optimised depth estimation models for resource-constrained autonomous driving perception systems [14]. Recently, the foundation model-based Depth Anything achieved robust zero-shot generalisation capabilities using large-scale unlabelled data, providing a robust technical foundation for this paper’s use of pseudo-point clouds to replace costly lidar [13].

Compared to international research focusing on foundational model architecture exploration, domestic studies concentrate more on specific industrial implementation scenarios. In recent years, with the deepening advancement of the intelligent manufacturing strategy, addressing the unique demands of cost control and process adaptability within the domestic manufacturing sector, Chinese scholars have conducted extensive research in the field of metal surface and weld defect detection. Regarding two-dimensional vision-based inspection, Li Zongyou et al. conducted a systematic review of existing deep learning detection methods, further confirming that solutions relying solely on two-dimensional images struggle to meet high-reliability inspection requirements when confronted with complex lighting variations and surface oil contamination in industrial settings [36].

To overcome the limitations of two-dimensional vision while reducing dependence on foreign high-end 3D equipment, exploring autonomous, controllable, and low-cost geometric depth acquisition solutions has emerged as a new research trend domestically. Lü Qinghai et al. proposed a three-dimensional vision-based inspection method for laser welding scenarios on power battery lids [37]. By constructing high-precision three-dimensional point cloud models of weld seams, they achieved precise segmentation of minute defects such as pits and blowholes using curvature features and region growing algorithms. Zhang Chen et al. addressed the demand for online laser welding inspection by employing line laser sensors to acquire high-density point cloud data. Combined with an enhanced deep learning algorithm, they achieved real-time three-dimensional reconstruction of weld morphology and defect identification [38]. However, most of these three-dimensional detection methods rely on expensive line laser scanners or structured light sensors, entailing high hardware costs and stringent installation calibration requirements. This makes large-scale deployment on low-cost production lines of small and medium-sized enterprises challenging.

Addressing the stringent constraints on algorithm real-time performance and hardware computational power for industrial deployment, lightweight network design remains a key focus in current domestic research. Xu Feihu et al. proposed a lightweight weld defect detection algorithm based on an enhanced MobileNetV3 architecture. By introducing an efficient channel attention mechanism, they substantially reduced model parameter counts while effectively enhancing focus on critical weld features, validating the attention mechanism’s efficacy in industrial quality inspection [39].

Synthesising these technological trends and inspired by domestic advances in 3D perception and lightweight design, this paper explores a novel detection paradigm balancing 3D high-precision with 2D low-cost advantages. Unlike costly 3D scanning approaches, we employ monocular depth estimation to generate pseudo-point clouds, combined with an enhanced dual-attention mechanism, to achieve low-cost, high-precision weld defect detection.

## 3. Materials and Methods

### 3.1. Dataset and Experimental Setup

To validate the effectiveness of the proposed method, the experimental dataset utilises authentic industrial welding defect data. The experimental dataset comprises normal welds and two typical structural anomalies: longitudinal and transverse missed welds. To develop a robust and generalized defect detection model, these two defect types were unified into a single ‘Defect’ category during the training and evaluation phases, thereby framing the problem as a binary classification task. The experiments employ the pseudo-point cloud generation workflow described in Section 4.2 to convert 2D images into 3D point cloud data. The dataset is randomly partitioned into training and validation sets at a 7:3 ratio. Furthermore, to ensure geometric consistency of the input data, a minimal number of low-quality samples were discarded during the pseudo-point cloud generation stage. Ultimately, 1635 valid 3D point cloud samples were retained for experimentation. Detailed category distribution and sample count statistics for the dataset are presented in Table 1.

All experiments were conducted on high-performance computing servers equipped with NVIDIA RTX A5000 professional-grade graphics accelerator cards (NVIDIA Corporation, Santa Clara, CA, USA) and Intel Xeon Platinum 8358P processors (Intel Corporation, Santa Clara, CA, USA). The system operating memory was 95 GB. The deep learning framework employed PyTorch 2.1.0 (PyTorch Foundation, San Francisco, CA, USA; https://pytorch.org/), with computational acceleration facilitated by CUDA 11.8 (NVIDIA Corporation, Santa Clara, CA, USA; https://developer.nvidia.com/cuda-toolkit, accessed on 20 May 2026). Network training utilised the Adam optimiser, with an initial learning rate set at 0.001. A cosine annealing strategy was introduced to dynamically adjust the learning rate, thereby preventing convergence to local optima. The batch size was set to 24, with a total training epoch count of 60. The number of points in the input point cloud was fixed at N = 1024 via farthest point sampling.

Given the extreme sensitivity of industrial quality inspection to missed defects, this paper employs overall accuracy, precision, recall, and F1-Score as evaluation metrics. Recall is the core metric of focus, as it reflects the model’s ability to capture defect samples and directly impacts industrial production safety. The calculation formulae are as follows:

(1) $$Recall=\frac{TP}{TP+FN}$$

where TP denotes correctly detected defects, and FN denotes missed defects.

### 3.2. Overall Framework

**Figure 1:**
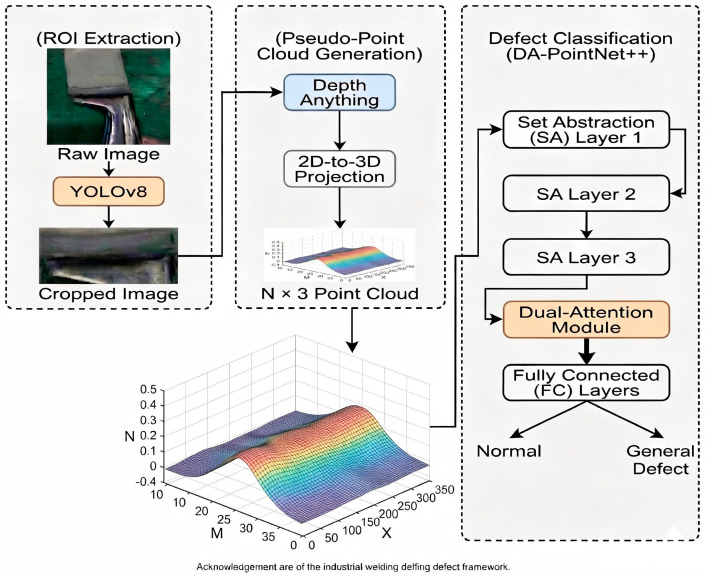
Overall Architecture of the Defect Detection Framework.

This paper proposes a coarse-to-fine industrial defect detection framework based on pseudo-point cloud generation, as illustrated in Figure 1.

The framework comprises three principal components: region of interest extraction, pseudo-point cloud generation, and a dual-attention-enhanced classification network:

1. Region of Interest Extraction: YOLOv8 is employed to rapidly localise and crop weld seam region images from complex backgrounds;
2. Pseudo-point cloud generation: The Depth Anything model infers monocular depth maps, which are then back-projected into 3D point clouds;
3. Geometric Feature Classification: Inputs the generated point cloud into a PointNet++ network incorporating dual attention modules for high-precision defect identification.

### 3.3. Pseudo-Point Cloud Generation

To acquire geometric information without costly 3D sensors, this paper employs monocular depth estimation techniques to construct pseudo point clouds. Given a cropped RGB image $I\in R^{H\times W\times 3}$, the pre-trained Depth Anything model Φdepth first infers the corresponding depth map D:

(2) $$D=\Phi_{depth}(I),\;D\in R^{H\times W}$$

**Figure 2:**
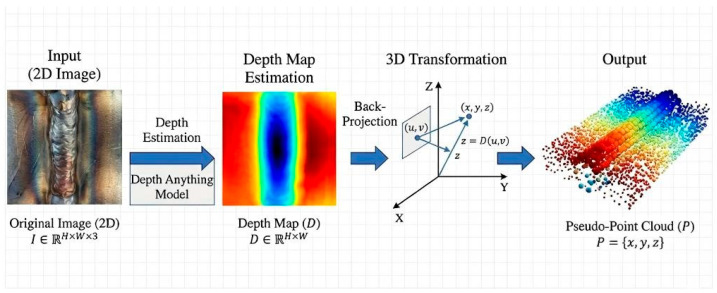
Pseudo-Point Cloud Generation Process.

Here, $D\left(u,v\right)$ denotes the depth value z at pixel coordinate $\left(u,v\right)$. Subsequently, the experiment assumes that the camera conforms to the pinhole imaging model and employs fixed camera intrinsic parameters to perform pseudo-point cloud backprojection. Each pixel point $\left(u,v\right)$ is backprojected into the three-dimensional Cartesian coordinate system $\left(x,y,z\right)$ using the camera intrinsic matrix K. The specific mapping formula is as follows:

(3) $$\begin{array}{l} x=\frac{(u-c_{x})\cdot z}{f_{x}}\\ y=\frac{(v-c_{y})\cdot z}{f_{y}}\\ z=D(u,v) \end{array}$$

where $\left(c_{x},c_{y}\right)$ denotes the principal point of the image and $\left(f_{x},f_{y}\right)$ represents the focal length. Following this transformation, the 2D image is converted into a point cloud set $P=\{p_{i}|i=1,...,N\}$ comprising N points, where $p_{i}=(x_{i},y_{i},z_{i})$. To adapt to network input requirements, the point cloud undergoes normalisation and is fixed to 1024 points via maximum point sampling. The complete visualisation workflow for this pseudo-point cloud generation is illustrated in Figure 2.

Input 2D weld seam image; depth map estimated using the Depth Anything model; generated 3D pseudo-point cloud containing geometric defect information.

**Figure 3:**
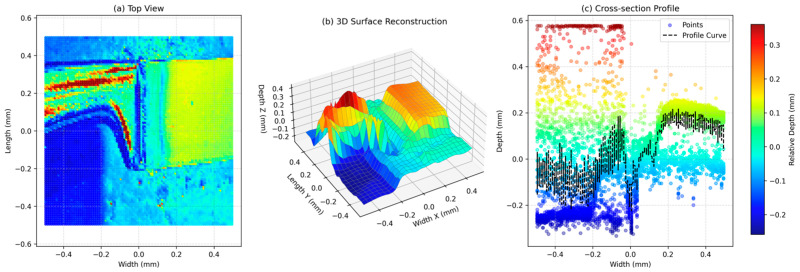
3D Pseudo-Point Cloud Reconstruction Visualization of Typical Solder Joint Defect Samples. (**a**) Depth heatmap top view, displaying the overall depth distribution; (**b**) 3D surface reconstruction view, where the meshed surface intuitively reveals the spatial structure of the weld surface; (**c**) X–Z cross-sectional contour along the centre of the defect region. The red contour lines clearly reproduce the geometric concavity of the missed weld defect, validating the effectiveness of monocular depth estimation.

To further validate the effectiveness of the aforementioned generation method in terms of geometric fidelity, we performed three-dimensional reconstruction visualisation on a genuine missed weld sample, as illustrated in Figure 3. Figure 3a displays the depth heatmap distribution from a top-down perspective; Figure 3b restores the spatial topology of the weld surface through surface reconstruction techniques, clearly demonstrating the continuity and smoothness of the point cloud data. Furthermore, Figure 3c illustrates the X–Z cross-sectional profile along the center of the defect region. As delineated by the red contour curve, the generated pseudo-point cloud captures a relative depth variation indicating a structural concavity, which correlates quantitatively with the physical morphology of the actual missing weld defect. This verifies that the monocular depth estimation pipeline effectively recovers relative spatial depth information essential for subsequent 3D topological classification.

It is worth noting that monocular depth estimation inherently introduces scale ambiguity. However, in our proposed framework, mapping to absolute physical dimensions (e.g., exact millimeters) is not strictly required. Since our primary objective is the geometric classification of topological anomalies rather than absolute dimensional measurement, all generated pseudo-point clouds are subjected to mean-centering and unit-sphere normalization during the data preprocessing stage. This scale-invariant transformation effectively circumvents the scale ambiguity challenge. Consequently, it forces the downstream DA-PointNet++ model to focus entirely on local structural variations and relative depth gradients—which are the core defining characteristics of weld defects—thereby ensuring highly robust classification performance.

### 3.4. DA-PointNet++ Network Architecture and Optimisationn

The backbone network selected for this study is based on PointNet++. Its core component is the Set Abstraction layer, which hierarchically extracts local features. For the input point set, the Set Abstraction layer first samples centre points via Farthest Point Sampling (FPS), then constructs local neighbourhoods using spherical queries. Assuming the features of a point within a local region are $f_{i}$, PointNet++ aggregates features through a multi-layer perceptron and max pooling:

(4) $$F_{local}=Max(MLP(f_{i}))$$

However, direct max pooling risks losing subtle geometric variations within local regions. To address this, a dual attention mechanism is introduced prior to feature aggregation.

To enhance the network’s perception of defect geometric orientation, this paper draws inspiration from CBAM’s design philosophy and proposes a dual attention module adapted for point clouds. This module consists of a series of channel attention and spatial attention.

1. Channel Attention

Channel attention aims to select feature channels exhibiting the strongest response to defects. Given an input feature map $F\in R^{N\times C}$, spatial information is first aggregated via global average pooling and global max pooling. Channel weights are then generated through a shared-weight MLP. The calculation formula for the channel attention map $M_{c}$ is as follows:

(5) $$M_{c}(F)=\sigma (MLP(AvgPool(F))+MLP(MaxPool(F)))$$

where σ denotes the Sigmoid activation function, and the MLP weights are shared between the two pooling outputs to reduce the number of parameters. The final refined feature map is $F\prime =M_{c}(F)⊗F$.

2. Spatial Attention

Following the reweighting of channel features, spatial attention is employed to localise critical defect points within the local neighbourhood. Average pooling and max pooling are performed separately on the channel dimensions. The resulting two 2D descriptors are concatenated and passed through a convolutional layer to generate the spatial weight map $M_{s}$:

(6) $$M_{s}(F\prime )=\sigma (f^{7\times 7}([AvgPool(F\prime );MaxPool(F\prime )]))$$

where $f^{7\times 7}$ denotes a convolution operation with kernel size 7×7. The final output feature F″ is computed as:

(7) $$F\prime \prime =M_{s}(F\prime )⊗F\prime$$

**Figure 4:**
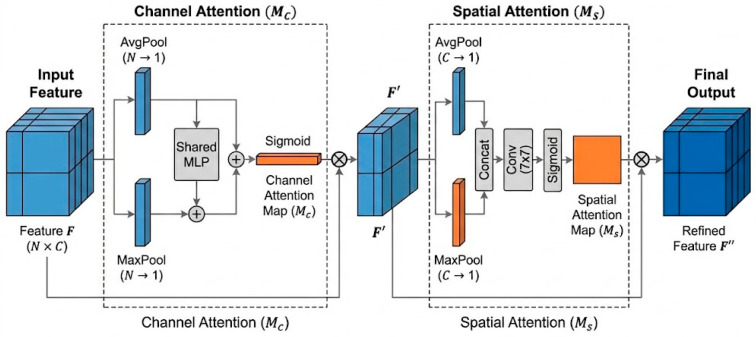
Schematic of the dual attention module.

Through this sequential dual-weighting mechanism, the network automatically focuses on regions of depth variation within local neighbourhoods, thereby achieving higher recall rates in experiments. The specific internal structure of this dual-attention module is illustrated in Figure 4.

This module captures subtle geometric details by sequentially aggregating features across both the channel and spatial dimensions. To drive supervised learning in DA-PointNet++ and achieve high-precision classification of weld defects, this paper constructs an optimised objective function for the network. Considering defect identification is fundamentally a binary classification task for general anomaly detection, a binary cross-entropy loss function is employed to measure the discrepancy between the predicted probability distribution and the true labels. This loss function is defined as follows:

(8) $$L=-\frac{1}{M}\sum_{i=1}^{M}\sum_{k=1}^{K}y_{i,k}\log({\hat{y}}_{i,k})$$

where M denotes the batch size, K represents the number of classes, y indicates the ground truth label, and $\hat{y}$ signifies the network’s predicted probability.

## 4. Experiments and Analysis

### 4.1. Comparative Experimental Results

To further evaluate the model’s learning efficiency and stability, we visualised a comparative analysis of the training processes between our proposed method and baseline models.

Benefiting from the dual attention mechanism’s rapid capture of critical geometric features, the proposed method exhibits exceptionally swift convergence during the initial training phase. To quantify this advantage, Table 2 further presents numerical values at key training milestones.

As presented in Table 2, distinct convergence and generalization dynamics are exhibited by the two models over a standardized training period of 60 epochs.

During the initial training phase, a faster initial convergence rate is demonstrated by the DA-PointNet++ model, which incorporates a dual-attention module. The performance metrics during the final stages of training reveal distinct convergence behaviors. While the baseline PointNet++ model reaches a training loss of 0.038 with a validation accuracy of 93.46%, the proposed DA-PointNet++ achieves a higher validation accuracy of 93.67% despite a plateaued training loss of 0.084. This dynamic suggests an implicit regularization effect provided by the dual-attention mechanism, which mitigates the tendency to overfit redundant background planar points and enhances generalization on unseen test samples. Due to a lack of attentional focus, the baseline model tends to overfit the abundant simple and redundant flat background point clouds within the training set, which leads to an artificially low training loss. Conversely, DA-PointNet++ compels the network to concentrate its optimization focus on the morphologically complex and difficult-to-fit topologies of minute defects. This mechanism effectively prevents the model from memorizing background noise, thereby yielding exceptional generalization capabilities when evaluated on unseen real-world test samples.

The experimental results are presented in Table 3. All comparison methods were trained and tested under identical dataset partitioning, input point counts, and training strategies to ensure fairness. Firstly, in terms of overall accuracy, the proposed method achieved 93.67%, marginally outperforming the baseline models PointNet++ (SSG) and PCT at 93.46%. Although the accuracy improvement is modest, it demonstrates the model’s robustness while maintaining high precision. More critically, our method exhibits significant advantages in addressing the most sensitive issue in industrial quality inspection: missed defects. DA-PointNet++ achieves a recall rate of 90.51%, representing a substantial 3.8 percentage point increase over the baseline PointNet++ (SSG) at 86.71%. This recall improvement demonstrates that incorporating the dual-attention mechanism enables the network to more effectively focus on the three-dimensional spatial distribution characteristics of defects. Consequently, it accurately detects both longitudinal and transverse missed welds that are visually similar yet prone to misclassification. Finally, when compared to a representative Transformer-based model, PCT achieves the same high accuracy of 93.46% as the baseline. However, its recall rate of 88.61% remains notably lower than that of the proposed method. This further demonstrates that the Dual-Attention module, specifically designed for local geometric features, is more targeted than the general global Transformer architecture when handling such subtle structural defects. It better meets the stringent industrial requirement of minimising the risk of missed detections.

Furthermore, to validate the superiority of the proposed method in geometric feature extraction under equivalent computational resources, a fair comparison was conducted between DA-PointNet++ and PointMLP, an advanced pure MLP architecture introduced in 2022, under a strict constraint of 60 training epochs. As presented in Table 3, although a certain level of defect recall is achieved by PointMLP after 60 training epochs, significant declines are observed in both its overall accuracy and macro-average F1-score, demonstrating a substantial performance gap compared to the proposed method.

A critical engineering fact is revealed by this performance contrast: when processing noisy monocular pseudo-point clouds, pure MLP models lacking local 3D geometric inductive biases tend to trade an increased false positive rate for higher recall, which results in a diminished comprehensive evaluation metric, the F1-score. In contrast, under the explicit guidance of the dual-attention mechanism, the topological structures of minute defects are rapidly isolated by DA-PointNet++ with exceptional efficiency. Moreover, an extremely low false positive rate is maintained, yielding the optimal overall F1-score of 92.06%. Consequently, in industrial deployment scenarios where the comprehensive control of missed detections and false alarms is highly prioritized, an absolute advantage for practical engineering implementation is demonstrated by the proposed method.

**Figure 5:**
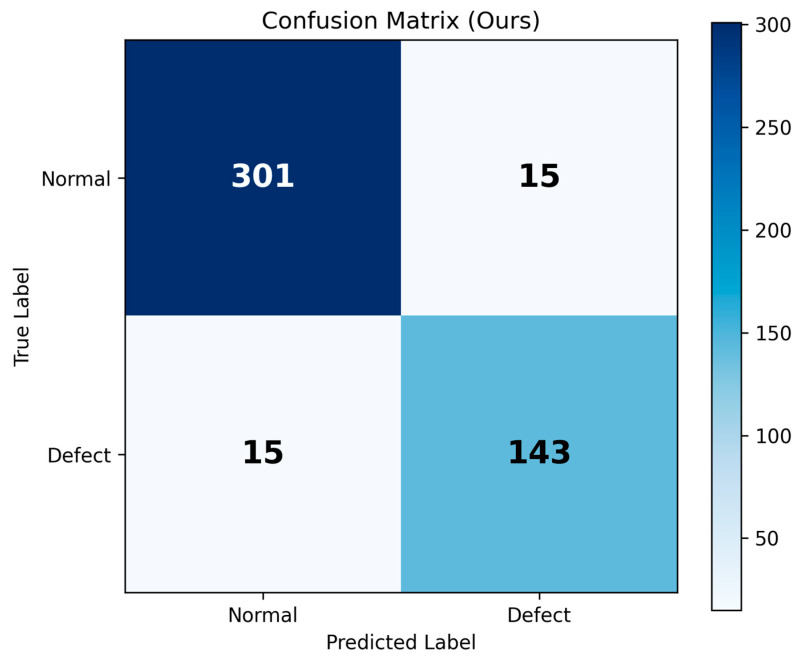
Confusion Matrix of DA-PointNet++ on the Test Set.

To further dissect the model’s specific classification behaviour across various sample types, we plotted the confusion matrix of DA-PointNet++ on the test set, as shown in Figure 5. The figure reveals a high-density distribution along the diagonal, indicating that the vast majority of samples were correctly classified.

Notably, addressing the critical issue of genuine defects being misclassified as normal in industrial quality inspection, our method produced only 15 false negatives among 158 defect samples. This achieves a high recall rate of 90.51% while effectively balancing false positive counts. This result intuitively demonstrates that the introduced dual attention mechanism can acutely detect subtle geometric anomalies, thereby significantly reducing the risk of severe structural defects escaping production lines.

### 4.2. YOLOv8 ROI Detection Performance

**Figure 6:**
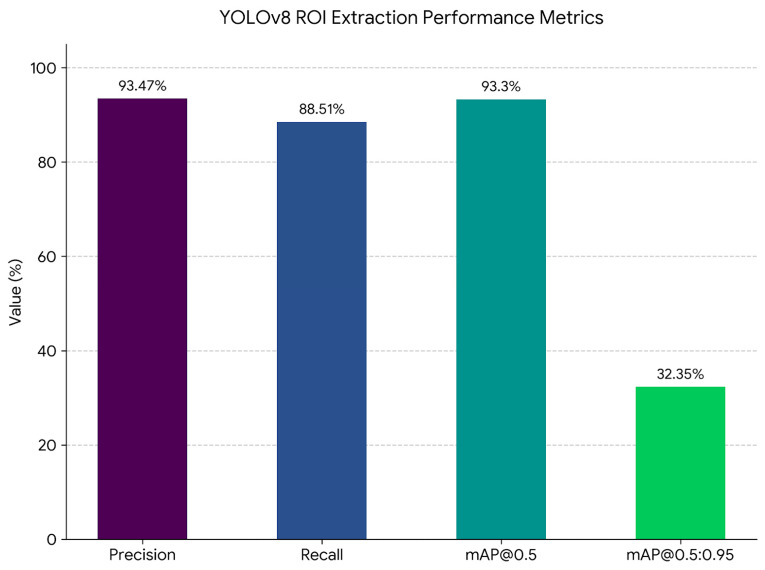
Quantitative performance metrics of the YOLOv8 model for front-end ROI extraction.

Before performing weld defect classification, the front-end YOLOv8 model must be capable of reliably localizing and extracting the target weld seam from complex industrial backgrounds, such as machinery, conveyors, and fixtures. The quantitative detection performance metrics of the trained YOLOv8 model on the test set are presented in Figure 6.

The front-end model demonstrates excellent coarse localization capability, achieving an mAP@0.5 of 93.30%, with a Precision of 93.47% and a Recall of 88.51%. Such high mAP@0.5 and Precision ensure that the vast majority of target weld seams are consistently and accurately bounded, while effectively avoiding excessive false positives from the background. It is worth noting that, due to the inherently ambiguous boundaries of industrial weld seams, the more stringent mAP@0.5:0.95 metric is relatively lower at 32.35%. Nevertheless, the high mAP@0.5 still clearly confirms that the target ROI has been successfully captured. Within the decoupled framework proposed in this study, the first stage primarily serves to eliminate non-weld background and provide focused input to the depth estimation network. Therefore, this reliable coarse localization fully satisfies the prerequisite requirements, does not constitute a system bottleneck, and lays a solid foundation for the subsequent 3D pseudo-point cloud generation.

### 4.3. System Efficiency and Complexity Analysis

In industrial laser cutting and welding scenarios, defect detection systems are typically subject to strict constraints imposed by the limited computational resources of edge devices. Therefore, evaluating the spatial and temporal complexity of a model is as critical as assessing its accuracy. To validate the lightweight design of the proposed method, a quantitative comparison was conducted between the baseline PointNet++ (SSG) and the proposed DA-PointNet++ in terms of the number of parameters (Params) and Floating-Point Operations (FLOPs).

As shown in Table 4, the baseline model requires 1.46 M parameters and 0.88 GFLOPs to process a single point cloud sample containing 1024 points. After incorporating the dual-attention module, the parameter count of DA-PointNet++ slightly increases to 1.59 M (an increase of approximately 8.95%), while the computational cost remains nearly unchanged at 0.88 GFLOPs.

This complexity analysis convincingly demonstrates that the proposed dual-attention mechanism achieves significant performance gains—particularly a 3.8% improvement in recall—while incurring almost no additional computational overhead. Moreover, it avoids the efficiency degradation commonly observed in computationally intensive Transformer architectures, further confirming that DA-PointNet++ is highly suitable for real-time edge deployment in low-cost manufacturing environments.

### 4.4. Robustness Analysis Against Environmental Noise and Geometric Perturbations

In practical industrial welding and laser cutting scenarios, sensor noise and strong specular reflections from metallic surfaces often lead to degraded image quality. These 2D visual disturbances inevitably propagate through the monocular depth estimation network, resulting in coordinate shifts or regional missing issues in the generated 3D pseudo point clouds. To evaluate the adaptability of the proposed DA-PointNet++ to such imperfect data, we conducted a 3D geometric robustness assessment on the test set.

Specifically, two types of geometric perturbations were introduced into clean point clouds:

1. Gaussian Jitter: Random noise sampled from a normal distribution N (0, 0.02) was added to the coordinates of each point to simulate micro-level depth estimation errors caused by insufficient illumination or sensor noise.
2. Random Dropout: 30% of the points in each point cloud were randomly removed, effectively simulating severe depth missing phenomena caused by specular overexposure on metallic surfaces.

The quantitative results are summarized in Table 5. Under continuous Gaussian jitter perturbations, the model demonstrated excellent stability, maintaining a high accuracy of 90.72% and a defect recall rate of 82.91%. Even under the extreme random dropout condition—where nearly one-third of the spatial geometric information was lost—DA-PointNet++ still achieved an accuracy of 79.54% and successfully detected 72.15% of the defects. The minimal performance degradation confirms that the embedded dual-attention mechanism enables the network to effectively capture the global topological features of weld defects, thereby demonstrating strong robustness and reliability for deployment in harsh industrial environments.

In actual industrial environments, the specular reflectivity of metallic weld surfaces and dynamic lighting conditions present significant challenges for traditional 3D sensors. To address this, the proposed DA-PointNet++ framework leverages the robust zero-shot generalization of the Depth Anything foundation model. Unlike active light sensors that are prone to ‘data holes’ due to specular highlights, our monocular approach relies on semantic geometric priors, enabling it to infer consistent depth maps even under complex reflective conditions.

The robustness experiments conducted in this section—specifically Gaussian Jitter and Random Dropout—serve as stress tests for these real-world variables. Gaussian Jitter (N = 0.02) simulates the pixel-level noise induced by fluctuating factory illumination, while the 30% Random Dropout directly emulates the phenomenon of severe depth information loss caused by specular overexposure on metallic surfaces. The fact that the model maintains a 79.54% accuracy and 72.15% recall even under 30% data loss demonstrates its exceptional resilience to the optical interference typically encountered in smart manufacturing.

### 4.5. Ablation Studies

**Figure 7:**
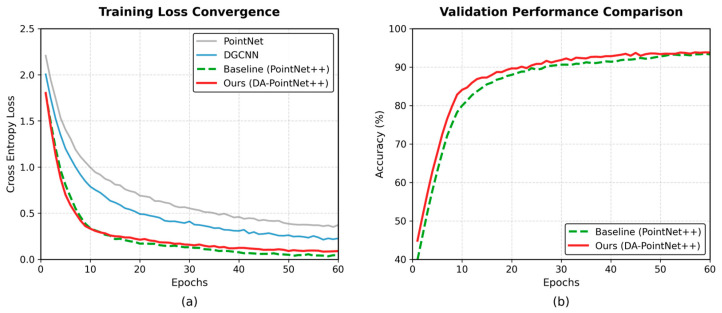
Training dynamics analysis. (**a**) Comparison of training loss convergence curves across different methods; (**b**) Dynamic Comparison of Validation Accuracy Between the Baseline Model and the Proposed Method.

To comprehensively evaluate the learning efficiency and training stability of the proposed model, and to visually compare its dynamic differences against the baseline model and other classical networks, the loss reduction trends and validation accuracy trajectories were recorded over a strictly standardized training period of 60 epochs. The visual comparison results are illustrated in Figure 7.

As illustrated by the training loss convergence curves in Figure 7a, DA-PointNet++ demonstrates an accelerated initial convergence. Specifically, within the first 20 epochs, the cross-entropy loss of the proposed method rapidly decreases to approximately 0.2, outperforming the baseline PointNet++ and DGCNN architectures. This accelerated optimization trajectory indicates that the explicit guidance of the dual-attention mechanism facilitates faster adaptation to critical 3D geometric features during the early training phase. Simultaneously, in contrast to the direct baseline model, overfitting to an abundance of redundant flat background points is circumvented by DA-PointNet++; instead, a healthy fitting lower bound is maintained. This intuitively demonstrates the implicit regularization effect of the dual-attention mechanism in enhancing the generalization capability of the model and isolating background noise.

Furthermore, the direct benefits of these healthy training dynamics on the validation accuracy are illustrated in Figure 7b. Throughout the entire 60-epoch evaluation of validation performance, the accuracy escalation of DA-PointNet++ not only commences from a higher initial point but also demonstrates enhanced stability against oscillations during the mid-to-late fine-tuning stages. Ultimately, the baseline model is consistently surpassed by the proposed method, securing the highest overall validation accuracy. This comprehensive dynamic evolution process sufficiently proves that the embedded dual-attention module not only elevates the upper limit of the network’s feature representation capabilities but also delineates a more efficient and robust optimization trajectory for the model within the underlying optimization space.

To validate the contributions of individual components within the dual attention module, ablation studies were conducted using PointNet++ (SSG) as the baseline, with the following variants: (A) adding only channel attention; (B) adding only spatial attention; (C) adding both dual attention components simultaneously.

Table 6 details the contribution analysis of each component within the dual attention module. Firstly, examining the impact of channel attention, introducing only the channel attention branch slightly improved the model’s accuracy by 0.64% compared to the baseline. This result indicates that by adaptively recalibrating feature channel weights, the network can to some extent suppress environmental noise unrelated to defects. Secondly, regarding the role of spatial attention, experiments revealed that employing spatial attention alone yielded more pronounced improvements in model performance. Notably, recall surged significantly from the baseline of 84.17% to 90.50%. This robustly confirms that the spatial attention mechanism guides the network to accurately detect critical geometric anomalies within sparse point clouds, thereby substantially reducing missed detections. Finally, regarding complementarity, integrating both mechanisms simultaneously yielded optimal performance in both accuracy and recall. This demonstrates that feature selection in the channel dimension and geometric localisation in the spatial dimension exhibit a complementary effect, jointly enabling the model’s high-precision identification of minute industrial defects.

To verify the stability and statistical reliability of the proposed DA-PointNet++, we conducted five independent replicate experiments using different random initialization seeds. As summarized in Table 7, the baseline PointNet++ achieved a mean Recall of 86.65% with a standard deviation of 0.31%. In contrast, our proposed DA-PointNet++ achieved a mean Recall of 90.48% with a remarkably tight standard deviation of only 0.18%. Furthermore, a paired *t*-test was conducted on the Recall metrics between the two models, yielding a *p*-value of p < 0.01. This highly significant statistical result confirms that the observed performance improvement is not due to random variance or lucky convergence. Instead, it robustly demonstrates that the Dual-Attention module consistently captures the critical topological features of weld defects, thereby ensuring highly reliable detection performance for industrial applications.

### 4.6. Visualisation of Feature Distribution and Attention Mechanism

To gain deeper insights into the model’s discriminative capability and decision-making basis within the feature space, this paper conducts visualisation analyses from two dimensions: macro-level feature distribution and micro-level regions of interest.

**Figure 8:**
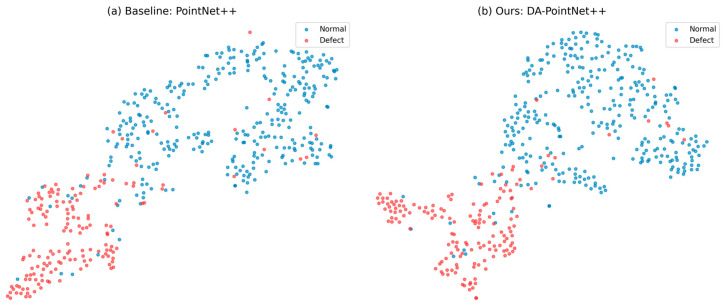
t-SNE visualisation comparison of test set feature embeddings. (**a**) The baseline model exhibits significant confusion at the decision boundary; (**b**) the proposed method achieves clear inter-class separation, demonstrating its capability to extract key geometric features.

First, to validate the dual attention module’s contribution to feature separability, we extracted feature vectors from test set samples prior to the fully connected layer and mapped them into a two-dimensional space using the t-SNE algorithm. As shown in Figure 8, the baseline PointNet++ model exhibits blurred feature boundaries when processing welds with complex surface textures, resulting in overlap between normal and minor defect samples in the feature space. In contrast, the dual-attention-enhanced DA-PointNet++ exhibits clear clustering with significantly increased inter-class distances. This high-distinctiveness feature distribution indicates the model successfully suppresses background noise to extract more robust geometric classification features.

**Figure 9:**
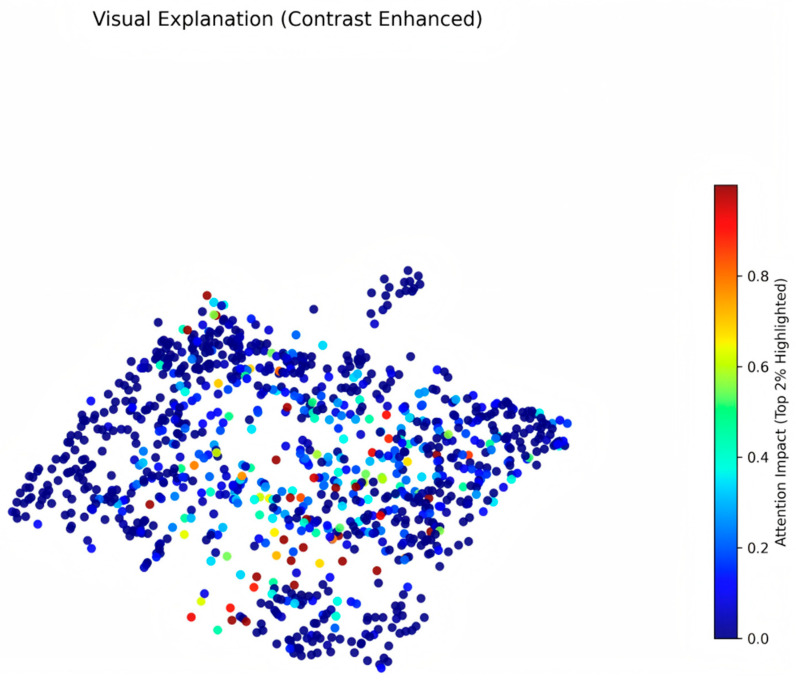
Visualization of feature attention weights of DA-PointNet++ on pseudo-point clouds.

To intuitively demonstrate the model’s effectiveness, visual analysis of the feature layer was conducted using Grad-CAM technology, with results shown in Figure 9.

As depicted, the intensity of the heatmap colours intuitively reflects the contribution of features to the classification decision. Red and yellow regions denote high-response zones intensively activated by the dual attention module, predominantly concentrated at locations of geometric discontinuities such as burrs and pores. Conversely, blue regions indicate effectively suppressed flat background areas and edge noise. The visualisation demonstrates that the dual attention mechanism directs the model’s focus towards regions exhibiting pronounced geometric variations within the point cloud, aligning with the observed trend of improved recall rates in preceding experiments.

### 4.7. Robustness Analysis Against Point Cloud Sparsity

In real-world industrial edge computing scenarios, stringent real-time requirements typically necessitate the further compression of the input point cloud scale via downsampling. To evaluate the robustness of the proposed DA-PointNet++ under extremely sparse inputs, a series of point cloud density ablation experiments were conducted. While maintaining other hyperparameters constant, the number of input points, N, was set to 256, 512, 1024, and 2048, respectively. The quantitative results are presented in Table 8.

As presented in Table 8, a positive correlation is generally observed between the overall accuracy and the number of input points, reaching 93.67% at N = 2048. However, this trend is not strictly followed by the recall rate of the defect category. Notably, when the number of points is halved from 1024 to 512, despite a marginal decrease of 0.84% in overall accuracy, a peak defect recall rate of 90.51% is achieved. The superiority of the proposed dual-attention module during the feature aggregation stage is robustly demonstrated by this counterintuitive phenomenon. Under the moderately sparse condition of N = 512, redundant flat weld background noise is more effectively filtered by the attention mechanism, thereby compelling the network to intensely focus on local anomalous geometric regions exhibiting significant abrupt depth changes.

Furthermore, even under the extremely sparse condition of N = 256, where nearly 75% of the geometric topological information is lost, an accuracy of 88.40% and a recall rate exceeding 80% are still maintained by DA-PointNet++. The immense potential of the proposed framework for low-latency and lightweight deployment on computationally constrained Industrial Internet of Things nodes is substantiated by this exceptional robustness against point cloud sparsity.

It is equally noteworthy that an identical defect recall rate is maintained when N = 1024 and N = 2048. This indicates that an optimal balance between geometric resolution and computational efficiency is achieved at N = 1024. The complete topological features of the defects are efficiently extracted by the proposed dual-attention mechanism as early as N = 1024, thereby rendering the additional points introduced at N = 2048 geometrically redundant for defect recognition.

### 4.8. System Complexity and Real-Time Inference Performance

In practical industrial laser welding inspection scenarios, inspection systems are typically deployed on edge devices with constrained computational resources and must be strictly synchronized with fast-paced production lines. Consequently, in addition to high classification accuracy, the spatial and temporal complexities of the model are equally critical metrics determining its deployment viability.

To comprehensively evaluate the execution efficiency of the proposed framework, a rigorous quantitative comparison between the baseline PointNet++ (SSG) model and the proposed DA-PointNet++ was conducted within a hardware environment equipped with a single NVIDIA RTX A5000 GPU. In terms of time measurement, to eliminate discrepancies induced by asynchronous GPU execution, a precise CUDA Event synchronization timing mechanism was employed, and adequate warm-up operations were executed. The testing condition was configured with a batch size of 1 to authentically simulate the stringent workflow of real-time single-frame inference encountered on industrial production lines. The quantitative evaluation results are presented in Table 9.

As presented in Table 9, when processing point cloud samples comprising 1024 points, the parameter count of DA-PointNet++, which incorporates the dual-attention mechanism, experiences only a marginal increase to 1.59 M, while its theoretical computational complexity (FLOPs) remains essentially constant at 0.88 G. During practical hardware inference, a single-frame latency of merely 3.24 ms is achieved, yielding a processing frame rate of up to 308.72 frames per second (FPS). This speed significantly exceeds the 30–60 FPS requirement of typical industrial cameras, demonstrating that the time overhead incurred during the 3D feature classification stage is minimal. Consequently, ample computational redundancy is preserved for the front-end YOLOv8 region extraction and monocular depth estimation tasks.

Furthermore, in conjunction with the aforementioned sparsity ablation experiments, when the scale of the input point cloud is further downsampled to N = 512, not only is a peak defect recall rate of 90.51% achieved by DA-PointNet++, but its single-frame inference latency is also further reduced to 2.99 ms, thereby elevating the FPS to 334.32. This exceptional characteristic, which concurrently ensures a low missed detection rate and high throughput, circumvents the computational bottlenecks commonly associated with Transformer-based architectures in point cloud processing. Consequently, the absolute engineering advantage of DA-PointNet++ for automated, low-cost industrial quality inspection production lines is firmly established.

### 4.9. Impact of ROI Detection Accuracy on Downstream Performance

To empirically validate the robustness of the downstream DA-PointNet++ classifier against upstream YOLOv8 bounding box inaccuracies, a controlled ROI boundary perturbation experiment was conducted. Instead of assuming perfect crops, artificial scaling and shifting perturbations at levels of 5%, 10%, and 15% were introduced to the original ROI images to simulate real-world bounding box errors. The perturbed ROIs were then processed through the depth generation and classification pipeline.

As shown in Table 10, the downstream Recall remains highly robust under 5% to 10% bounding box errors (dropping by only 0.71% and 2.01%, respectively). This tolerance range effectively covers the vast majority of suboptimal predictions encountered in the 2D detection stage. Performance degrades significantly only when the ROI boundary error reaches 15%, a level at which the actual topological structure of the weld defect begins to be physically cropped out of the image. This experiment conclusively demonstrates that the proposed decoupled framework relies on the robust internal geometric structures of the pseudo-point clouds rather than perfect bounding box edges, fully justifying the system’s practical reliability.

## 5. Discussion

The results of this study demonstrate that the proposed DA-PointNet++ model achieves an overall accuracy of 93.67% and a high recall rate of 90.51% in the unified binary weld defect detection task. This indicates that extracting 3D geometric features via pseudo-point clouds effectively overcomes the intrinsic limitations of traditional 2D optical image detection, which is highly susceptible to complex environmental lighting, oil interference, and low-contrast surface textures. Compared to conventional industrial 3D scanning equipment that is costly and demands strict installation calibration, our framework utilizes the Depth Anything model for zero-shot monocular depth estimation, providing a highly reliable geometric representation without relying on expensive hardware.

Furthermore, compared to mainstream point cloud networks such as PointConv, DGCNN, and the Point Cloud Transformer, our DA-PointNet++ exhibits a distinct advantage in minimizing missed detections. While PCT achieved a competitive accuracy, its recall rate was significantly lower than that of our method (88.61% vs. 90.51%). This performance enhancement is primarily attributed to the embedded dual-attention module. By cascading channel and spatial attention mechanisms, the network successfully filters out background noise and precisely localizes structural mutations within local neighborhoods. Visual explanations using t-SNE and Grad-CAM further corroborate that the dual-attention mechanism enables the model to focus highly on geometric anomaly regions corresponding to longitudinal and transverse missed welds, thereby significantly reducing the risk of these critical structural defects escaping the production line. Ultimately, this 2D–3D collaborative detection framework offers a highly cost-effective paradigm for automated surface quality control in smart manufacturing.

Despite the substantial advantages demonstrated, this study has several limitations that warrant objective consideration:

- Data Scale: The current experiments were validated on a dataset of 1635 valid 3D point cloud samples. Expanding the data scale in future research could further enhance the model’s robustness.
- Environmental and Methodological Applicability: Although the pseudo-point cloud strategy reduces hardware costs, monocular depth estimation models may still encounter challenges such as local geometric distortions when facing extreme lighting conditions or highly reflective and textureless welding surfaces.
- Experimental Condition Constraints: The proposed framework currently employs a decoupled, multi-stage strategy (ROI extraction, pseudo-point cloud generation, and classification). While this modular design facilitates independent component upgrades in industrial settings, it inherently introduces the risk of cumulative errors across stages. Furthermore, although the DA-PointNet++ backend is extremely lightweight and fast, the overall system’s inference latency is currently bottlenecked by the Depth Anything foundation model due to its relatively large parameter size (e.g., 24.8 M for the small variant). Future research will focus on end-to-end network integration and applying knowledge distillation techniques to compress the depth estimation module, thereby further elevating the holistic throughput of the inspection system.

Future research directions should address these limitations. On one hand, efforts will focus on exploring a differentiable end-to-end joint optimization architecture to unify depth estimation and point cloud classification. Additionally, the exploration of model pruning and quantization techniques represents a viable future direction to further minimize inference latency, potentially facilitating broader lightweight deployment on resource-constrained industrial edge devices.

## 6. Conclusions

This study establishes a 2D–3D collaborative detection framework that effectively balances high-precision geometric perception with low hardware dependency for industrial weld defect inspection. To address the high deployment costs of traditional 3D sensors and the limited robustness of 2D visual inspection, the framework innovatively integrates YOLOv8 with the Depth Anything model to generate high-fidelity 3D pseudo-point clouds from single RGB images. Furthermore, a lightweight DA-PointNet++ architecture is designed by embedding a dual-attention module into the backbone network. This mechanism adaptively recalibrates features across channel and spatial dimensions, significantly enhancing the network’s perception of spatially oriented structural anomalies. Extensive experiments on a real-world welding dataset demonstrate that the proposed method achieves an accuracy of 93.67% and a critical recall rate of 90.51%. By substantially reducing the missed detection rate while maintaining low computational overhead, this 2D–3D collaborative approach successfully balances high precision with low hardware dependency, offering a highly practical and scalable solution for automated quality inspection in smart manufacturing.

## Figures and Tables

**Table 1:** Detailed category distribution statistics for the weld defect dataset.

Dataset	Total Number of 3D Image Samples	No Defect	Weld Defect (Mixed)
Training set	1161	772	389
Validation set	474	316	158
Total	1635	1089	546

**Table 2:** Quantitative comparison of model training convergence characteristics and final performance metrics.

Method	Epoch 10 (Loss)	Epoch 30 (Loss)	Final Loss (Ep. 60)	Final Accuracy (%)
Baseline (PointNet++)	0.339	0.129	0.038	93.46
Ours (DA-PointNet++)	0.329	0.159	0.084	93.67

**Table 3:** Performance comparison of different methods on the welding defect dataset.

Method	Year	Input	Accuracy (%)	Recall (%)	F1-Score (%)
PointNet++	2017	Point	90.08	86.71	88.35
PointNet++ (SSG)	2017	Point	93.46	86.71	90.97
PointNet++ (MSG)	2017	Point	93.25	87.34	90.20
DGCNN	2019	Point	92.83	89.87	91.32
PointConv	2019	Point	92.54	89.24	90.15
PCT	2021	Point	93.46	88.61	90.97
PointMLP	2022	Point	91.77	87.97	90.92
Ours (DA-PointNet++)	2025	Point	93.67	90.51	92.06

**Table 4:** Method complexity comparison between the baseline and the proposed DA-PointNet++.

Method	Params (M)	FLOPs (G)
Baseline (PointNet++)	1.46	0.88
Ours (DA-PointNet++)	1.59	0.88

**Table 5:** Robustness evaluation of DA-PointNet++ under different 3D geometric perturbations.

3D Noise Condition	Accuracy (%)	Recall (Defect) (%)
Clean (No Noise)	93.67	88.87
Gaussian Jitter	90.72	82.91
Random Dropout (30%)	79.54	72.15

**Table 6:** Ablation study results for the components of the dual-attention module.

Method Variants	Channel Attn	Spatial Attn	Accuracy	Recall
Baseline (PointNet++)	×	×	90.92	84.17
Channel Only	√	×	91.56	84.17
Spatial Only	×	√	93.03	90.50
Ours (DA-PointNet++)	√	√	93.67	90.51

**Table 7:** Statistical significance analysis over five independent runs.

Model	Mean Accuracy (%)	Mean Recall (%)	p-Value (*t*-Test on Recall)
PointeNet++ (Baseline)	93.42 ± 0.15	88.87	-
DA-PointNet++ (Ours)	93.65 ± 0.12	82.91	p<0.01

**Table 8:** Effect of Different Numbers of Input Points N on Model Performance.

Input Points (N)	Accuracy (%)	Recall (Defect) (%)
2048	93.67	89.87
1024	93.46	89.87
512	92.62	90.51
256	88.40	81.65

**Table 9:** Evaluation of System Complexity and Real-Time Performance.

Model	Input Points	Params (M)	FLOPs (G)	Latency (ms)	FPS (Frames/s)
PointNet++ (Baseline)	1024	1.46	0.88	3.51 ± 0.01	284.77
Ours (DA-PointNet++)	1024	1.59	0.88	3.24 ± 0.10	308.72
Ours (DA-PointNet++)	512	1.59	0.44	2.99 ± 0.01	334.32

**Table 10:** Impact of controlled ROI boundary perturbations on downstream classification performance.

ROI Perturbation Level	Accuracy (%)	Recall (%)	Recall Drop (%)
0% (Ground Truth Crop)	93.67	90.51	-
5% Shift/Scale Error	93.05	82.91	0.71
10% Shift/Scale Error	91.82	88.50	2.01
15% Shift/Scale Error	86.40	83.20	7.31

## Data Availability

The 2D image data used in this study were obtained from on-site photography at the factory.
